# Immunocytochemical study of canine lymphomas and its correlation with exposure to tobacco smoke

**DOI:** 10.14202/vetworld.2017.1307-1313

**Published:** 2017-11-07

**Authors:** K. C. Pinello, M. Santos, L. Leite-Martins, J. Niza-Ribeiro, A. J. de Matos

**Affiliations:** 1 Department of Veterinary Clinics, Institute of Biomedical Sciences Abel Salazar, University of Porto, Porto, Portugal; 2 Department of Microscopy, Institute of Biomedical Sciences Abel Salazar, University of Porto, Porto, Portugal; 3 Department of Population Studies, Institute of Biomedical Sciences Abel Salazar, University of Porto, Porto, Portugal; 4 EPI Unit, Institute of Public Health, ISPUP – University of Porto, Porto, Portugal; 5 Animal Science and Study Centre, Food and Agrarian Sciences and Technologies Institute, University of Porto, Porto, Portugal

**Keywords:** canine lymphoma, immunocytochemistry, proliferation, tobacco smoke

## Abstract

**Aim:**

Canine lymphoma is one of the most common canine neoplasms, but little is known regarding the effects of exposure to tobacco smoke on their biologic behavior. As cytology is the most frequent diagnostic method of canine lymphoma, the aims of this study were to perform an immunocytochemical study of canine lymphomas, including subtyping and cell proliferation analysis, and to establish their correlation with tobacco smoke exposure.

**Materials and Methods:**

A total of 23 dogs diagnosed with lymphoma were subjected to careful fine-needle biopsies of enlarged lymph nodes. The smears were air-dried, fixed with cold acetone, and immunocytochemically stained using CD3, PAX5, and Ki-67. Owners were requested to complete an epidemiologic questionnaire.

**Results:**

According to the updated Kiel classification, 65% were B-cell lymphomas - three low grade (LG) and 12 high grade (HG) and 35% were T-cell -two LG and six HG. Thirteen tumors presented high Ki67 indexes (>40%) (11 HG and 2 LG), two revealed moderate ones (20-40%) (1 HG and 1 LG), and three had low indexes (≤20%) (1 HG and 2 LG). Both a significant positive correlation and a significant linear-by-linear association (p=0.018) were observed between high Ki67 indexes and smoking owners (r=0.753, p=0.002) as well as with the number of smokers in the household (r=0.641, p=0.001). Moreover, the mean percentage of Ki67^+^ cells from the group of “smoker owners” was statically higher (p=0.011) than that from the “non-smoker owners.”

**Conclusion:**

The results suggest that cytological diagnosis of canine lymphomas benefits from being complemented with immunocytochemical studies that include subtyping and assessment of proliferative activity, both contributing for the prognosis and therapeutic planning. Furthermore, exposure to tobacco smoke seems to be related to the biological behavior of canine lymphomas.

## Introduction

Lymphomas are a wide and motley group of neoplasms which includes several subtypes with distinct clinical and epidemiological characteristics [1]. They represent one of the most common neoplasms of the dog, comprising approximately 7-24% of all neoplasms in this species [2]. The increasing frequency of canine lymphomas [2,3] mimics a trend in human oncology in which non-Hodgkin’s lymphoma (NHL) represents 5% of all new cancer cases, currently being one of the top five leading cancer-related deaths [4]. Dogs and humans are close companions and thus subjected to similar indoor and outdoor environmental influences. Therefore, epidemiological studies using dogs as sentinels provide an opportunity to assess the effects of environmental factors on the biologic behavior of cancer [5,6].

Contrary to human medicine, and often due to financial constraints, cytology plays a key role in the diagnosis of canine lymphoma [3]. The cytological analysis is a suitable, easy, and inexpensive diagnostic method, which is in agreement with the histological classification of most subtypes [3]. According to our experience, more than 70% of lymphoma diagnoses in dogs are achieved through cytology (unpublished data). Arecent publication [7] calculated that 90% of all canine lymphoma diagnoses were obtained using cytology. Although immunocytochemistry (ICC) [8] was studied in the past decades, it has not been routinely included in the diagnostic approach of canine lymphomas [9,10].

The updated Kiel classification adapted to the canine species [11] is the most frequently used classification scheme for the cytological diagnosis of canine lymphomas [9,11-13]. Lymphomas are essentially classified on the basis of cell morphology and immunological characteristics and subtyped according to the fundamental distinction between phenotypes (B or T) and malignancy grades (low or high)[13]. Such evaluations allow for a 90% accurate characterization [5,10,14]. As in human medicine, the classification of lymphomas is an important prognostic tool [12,15,16] since, if left untreated, high-grade (HG) lymphomas have significantly higher mortality rates than low-grade (LG) ones [17]. Yet, the former responds better to aggressive chemotherapeutic protocols, sometimes even achieving complete remissions[18]. There are, however, variations in survival rates within similar subtypes and grades, suggesting that an individual accurate prognosis may benefit from additional evaluations, such as the analysis of proliferative activity at the time of diagnosis [9,18].

Epidemiological studies of companion animals have been increasing, defining the dog as a sentinel[19] of potential risk factors for human health, mainly due to shared environments, shorter disease latency, and spontaneous diseases as lymphomas[6,20-23]. Several environmental and lifestyle factors were associated with human NHL [24], and these seem to influence both the overall and subtypes incidences[1,25]. One of these factors is the exposure to cigarette smoking, which represents a risk factor primarily for follicular lymphomas in human patients[26]. Some studies have shown different risk factors for the development of canine lymphomas such as breed [27], sex and hormonal exposure[28], *Bartonella* infection [29], oxidative stress [30], household chemicals [6,31], herbicides [32], magnetic fields[33], and air pollution [5,19-21,34]. It is noteworthy, though, that no environmental factors were related to the biological behavior of spontaneous canine lymphomas.

Epidemiological data evidenced a correlation between tobacco smoking and numerous human cancers [35]. Furthermore, new insights emerged regarding second-hand smoking, also named “environmental tobacco smoke” (ETS), “passive smoking,” or “involuntary smoking” [36], as well as third-hand smoking (THS) [37]. ETS consists on inhaling both the smoke exhaled by smokers and the sidestream smoke produced by the burning cigarette that contains the same carcinogenic compounds inhaled during active smoking [38]. In addition, when someone smokes, several nicotine-like contaminants are exhaled and settle down on various household surfaces and objects (carpets, table tops, utensils, and furniture), remaining there for weeks or months [37]. These are later reemitted in a gas phase, reacting with oxidants and other environmental compounds, a phenomenon that characterizes the THS [37]. Companion animals are undoubtedly second-hand smokers [39], and most certainly, third-hand smokers when sharing the household with smoking owners. As “active sniffers,” dogs are even more exposed to a wide range of potential chemical carcinogens that easily surpass the exposure to which human adults or children are subjected.

Although human studies suggested a positive association between tobacco exposure and the risk of follicular NHL [38], veterinary studies on the effects of tobacco smoke on lymphomas are inexistent.

The aim of this study was to assess the role of the immunocytochemical evaluation of canine lymphomas in the improvement of current routine diagnosis. Furthermore, another goal was to identify associations between exposure to several environmental factors, including exposure to tobacco smoke, lymphoma subtypes, and their biological behavior.

## Materials and Methods

### Ethical approval

The study was approved by the Bioethics Committee of the Institute of Biomedical Sciences Abel Salazar of the University of Porto (ORBEA-ICBAS-UP).

A prospective series of 23 dogs with a cytological diagnosis of lymphoma was considered. From each dog, lymph node cytological specimens were collected for diagnostic purposes. Owners were informed about the aims and methods of the study and signed an informed consent form. Dogs that had received previous chemotherapy were excluded from the study.

### Immunocytochemistry

Fine-needle aspiration biopsies, or just fine-needle biopsies (FNB), were obtained using a 21-gauge needle without aspiration. The material inside the needle was immediately smeared using a 10ml disposable plastic syringe and air-dried. Aminimum of six smears per enlarged lymph node were produced. Submandibular lymph nodes were avoided, whenever possible because their drainage of the oral cavity consistently yields marked hyperplastic features [40]. For diagnostic purposes, a minimum of two smears were stained with Hemacolor (Merck, Darmstadt, Germany). Lymphoma was diagnosed when the normal heterogeneous population of lymphocytes was replaced by predominantly immature and/or monomorphic lymphoid cells [41,42].

For the immunocytochemical assays, smears were dried, fixed in cold acetone for 2min, stored at 2-6°C, and processed within 48h. Immunocytochemical stains were conducted with commercially available antibodies: The pan T-lymphocyte marker (polyclonal rabbit antihuman) CD3 (Dako, Glostrup, Denmark) for T-lymphocytes, monoclonal mouse anti-PAX5 antibody (Leica Biosystems, Nussloch, Germany)[43] for B-lymphocytes, and anti-Ki67 monoclonal antibody (MIB-1 mouse) (Dako, Glostrup, Denmark) for proliferation assessment. Immunocytochemical procedures were performed according to the manufacturers’ protocols (Novolink Polymer^®^; Leica Microsystems, Nussloch, Germany), using the indirect immunoperoxidase staining technique. Heat-induced epitope retrieval was conducted in a hot water bath (100°C, 23min) by incubating slides in 10 mM citrated buffer (pH6.0). The slides were then gradually cooled to room temperature. After being washed in 0.05 M tris-buffer (pH7), the slides were incubated with the protein block for 5min. Each section was then incubated for 120min with primary antibodies diluted in 10% bovine serum albumin (Sigma, Darmstadt, Germany) (CD3 and Ki671:50; PAX51:40) at room temperature. The endogenous peroxidase activity was neutralized using the peroxidase block, and slides were then incubated with the second antibody (rabbit anti-mouse IgG) for 20min. Diaminobenzidine was used as a chromogen, and slides were counterstained with Mayer’s hematoxylin. Smears of normal spleen were used as positive controls. The percentage of positive cells was determined by counting 300cells at ×400 magnification in selected fields that showed good cell preservation. All cells with the expected staining pattern (membrane/cytoplasmic for CD3 and nuclear for PAX-5 and Ki67) were considered positive, regardless of the staining intensity. The immunophenotype of each lymphoma was based on the most prevalent number of positive cells [43]. For the proliferation assessment, lymphomas were classified, according to the number of positive Ki67cells, as follows: Low index (≤20% Ki67^+^ cells), moderate index (20-40% Ki67^+^ cells), or high index (>40% Ki67^+^ cells) as previously described [15].

Tumors were classified according to the updated Kiel classification adapted to the canine species [9], based on cell size and shape; cell volume and intensity of cytoplasm staining; nuclear size, shape, and position; nucleoli size, distinctiveness, number and positioning; appearance of nuclear chromatin; and mitotic indexes (MIs) [44].

### Exposure and covariate assessment

Owners were requested to answer a 70-question questionnaire about their dog’s demographics, health-related characteristics, and environmental exposures. Questions regarding the animal included age; sex; breed; reproductive status; date and origin of acquisition (shelter, breeder, pet shop, stray, or other); hair length; nose length; food type; weight; body type; grooming care; use of flea/tick control products; and physical activity levels. Environmental factors included household smoking; house size; use of carpets, rugs, and curtains; house location; the amount of time the dog spent outdoors; the use of fireplace; and exposure to environmental chemicals such as herbicides, pesticides, fungicides, rodenticides, and cleaning products such as bleach and ammonia. When applicable, the questionnaire included exhaustive details about exposure to tobacco smoke and its circumstances, the number of former or current smoking cohabitants, the number of daily consumed cigarettes (or similar), and the length of smoking time during the past decade.

### Statistical analysis

Data were analyzed with the Statistical Package for the Social Sciences (SPSS), version24. Apairwise comparison with all the collected variables was performed, and the significance of the Spearman’s rank correlation coefficient was established. The difference between the average percentage of positive Ki67cells (%Ki67^+^) in the groups “smoker owners” and “non-smokers owners” was analyzed with one-way ANOVA. The linear-by-linear association between two levels of smoke exposure and three levels of Ki67-low, moderate, or high, previously described-was tested with the Chi-squared test. For all tests, a two-sided p<0.05 was considered to be statistically significant.

## Results

### Classification

**Figure-1:**
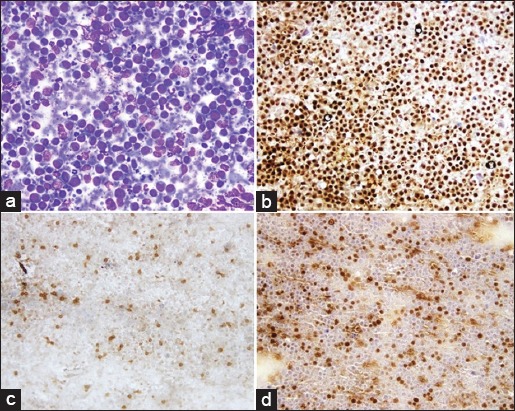
B-cell centroblastic/centrocytic lymphoma of a 3-year-old mongrel female dog. Fine-needle aspiration biopsies of enlarged pre-scapular lymph node. (a) A population of medium-to-large lymphoid cells admixed with very few mature lymphocytes Hemacolor, ×400; (b) PAX5 immunostain (×200); (c) CD3 immunostain (×200); (d) Ki67 immunostain - moderate index: 40% (×200).

According to the updated Kiel classification, 65% (15/23) were B-cell and 35% (8/23) were T-cell lymphomas. Eight morphological subtypes were identified including five B-cell and four T-cell subtypes. Out of the B-cell ones, three were LG centroblastic/centrocytic (Figure-1) and 12 HGs: One centroblastic large cell, five centroblastic monomorphic, four centroblastic pleomorphic, and two immunoblastic. Out of the eight T-cell lymphomas, two were LG small clear cell, and six were HG: One pleomorphic large cell, one lymphoblastic, and four pleomorphic mixed (Table-1).

**Table-1:** Updated Kiel classification, percentage of Ki67-positive cells and Ki67 indexes, and household smoking status of canine lymphomas.

Kiel classification	%Ki67^+^	Ki67 Index	Smoker owners	Number of smokers
**B-cell lymphomas**				
**High grade**				
Centroblastic large cell	48	High	Yes	2
Centroblastic monomorphic	88	High	Yes	1
Centroblastic monomorphic	52	High	Yes	2
Centroblastic monomorphic	51	High	-	-
Centroblastic monomorphic	-	-	Yes	1
Centroblastic monomorphic	-	-	No	0
Centroblastic pleomorphic	31	Moderate	Yes	1
Centroblastic pleomorphic	89	High	-	-
Centroblastic pleomorphic	95	High	-	-
Centroblastic pleomorphic	-	-	Yes	2
Immunoblastic	83	High	Yes	1
Immunoblastic	90	High	Yes	1
**Low-grade**				
Centroblastic/centrocytic	42	High	Yes	1
Centroblastic/centrocytic	20	Low	No	0
Centroblastic/centrocytic	40	Moderate	No	0
**T-cell lymphomas**				
**High-grade**				
Lymphoblastic	8	Low	No	0
Pleomorphic mixed	87	High	Yes	2
Pleomorphic mixed	61	High	Yes	1
Pleomorphic mixed	56	High	No	0
Pleomorphic mixed	-	-	No	0
Pleomorphic large cells	-	-	No	0
**Low-grade**				
Small clear cell type	85	High	-	-
Small clear cell type	20	Low	No	0

### Proliferation assessment

Ki67 ICC was performed in 18cases with well-preserved cytological smears. The mean % Ki67^+^ was 58.1% (standard deviation [SD]=0.28), and the median was 54% (range 8-98%). Thirteen lymphomas were categorized as high proliferation index (X_=0.71; SD=0.19), 11 of them being HG ones and two were LG. Two lymphomas presented moderate proliferation indexes (X¯=0.35; SD=0.06), and three cases presented low Ki67 indexes (X¯ =0.16; SD=0.69) (Table-1).

### Epidemiological study

The series included seven (30.4%) mongrel and 16(69.6%) purebred dogs (six labrador retrievers-26%, two boxers-8.7%, and one each of pointer, Portuguese hunting dog, bulldog, cocker spaniel, golden retriever, Castro Laboreiro shepherd, and pit bull terrier). Eleven dogs (47.8%) were females (45% spayed), and 12(52.2%) were males (8% castrated). The mean age was 8.1years (range, 3-15years).

The epidemiologic questionnaire was completed by all dog owners although only 19 answered the section concerning smoking habits (Table-1). The results showed that in 11 out of 19cases (57.89%) either the owners or other cohabitants, from 2005 to 2016, were or had been active smokers. In seven cases (63.7%), the dog lived with one smoker, while in the remaining cases (36.3%), there were two smokers cohabiting with the dog.

**Figure-2:**
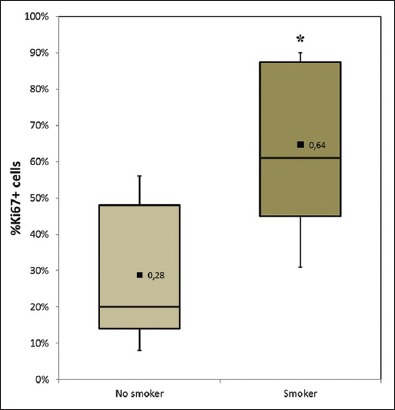
Box-and-whisker diagram depicting the percentage of Ki67^+^ cells categorized by the smoking status. Represents the mean percentage of Ki67^+^ cells per group. Lymphomas affecting dogs owned by smokers have a significantly higher mean percentage of Ki67^+^ cells (p<0.05).

Pairwise comparison between all studied variables revealed a statistically significant positive correlation (r=0.753, p=0.002) and significant linear-by-linear association (p=0.018) between the Ki67 index and smoking status. Data also revealed a statistically significant positive correlation between the Ki67 index and the number of smokers who lived with the dog (r=0.641, p=0.018). The ANOVA test confirmed a significantly different (p=0.011) mean percentage of Ki67^+^ cells between the groups “non-smoker owners” (X_ =0.25; SD=0.19) and “smoker owners” (X_ =0.64; SD=0.22) (Figure-2). Neither other environmental factors nor geographical localization revealed significant relations with subtype, grade, or tumor proliferation index.

## Discussion

Since lymphomas are a wide group of hematopoietic neoplasms with a broad range of potential outcomes [2,7,16], the simple diagnosis of “lymphoma” is of limited value for the clinical management of patients or even for the conduction of epidemiological studies. ICC plays a unique role in the improvement of the cytological diagnosis of canine lymphoma.

As lymphoid tissues lack in cellular cohesion, they permit a rich cellular harvest, so well-executed cytological collection procedures followed by adequate processing allow for obtaining useful diagnostic information [45]. In our study, FNB was performed without suction (the Zajdela or “French” technique)[8,46]. Although this technique potentially reduces the amount of harvested material, the preparations are more representative, less contaminated with blood and other cells [8] and cells are better preserved, which indeed contributes for the quality of immunocytochemical analysis.

Lymphoma cytological subtyping is challenging, and the use of ICC plays a vital role in both the diagnosis and subtyping [8]. Although all lymphoma immunophenotyping techniques-immunohistochemistry, flow cytometry, and PARR-have their particular advantages [8], ICC allows for morphological evaluation of stained cells as well as staining location and intensity in a simple smear, thus reducing costs and procedures.

MI and Ki67 immunolabeling are the proliferation parameters more commonly used in veterinary oncology [7]. Counting the number of mitotic figures in cytological preparations can be misleading and lacks representativeness, mainly due to an irregular cell distribution [7]. The ICC for Ki67, a non-histone nuclear protein expressed in all stages of the cell cycle, may overcome this difficulty and thus improve the assessment of the proliferation activity of lymphomas. It has been shown that the Ki67 immunolabeling of cytological smears had high concordance with Ki67 immunohistochemistry in biopsy tissues [9]. In this study, we opted to count 300cells per tumor, a higher number than previously reported (100cells), to ensure the reliability of the results [7,47].

In our series, there was a higher prevalence of B-cell (65%) over T-cell lymphomas (35%), and 56.5% were HG tumors (13/23). Interestingly, Ki67 index differences were detected within tumors of similar grades, both in B-and T-cell lymphomas. There was no statistically significant correlation between Kiel’s grades and Ki67 indexes or the percentage of Ki67-positive cells. Considering that cytotoxic chemotherapy is the preferred treatment for dogs with lymphoma and that it is known that it yields better responses in highly proliferating tumors, it is possible that subtyping and grading fail as prognostic and predictive indicators if not complemented with proliferation markers. In human mantle-cell lymphoma, the Ki-67 index has already surpassed cytology and growth pattern as prognostic factors [48]. Moreover, survival time discrepancies within canine lymphoma subtypes have already been demonstrated by Ponce *et al*. [12]. In a recent study [15] of HG B-cell lymphomas treated with a modified Wisconsin-Madison protocol, dogs affected by tumors of intermediate Ki67% indexes presented longer survival and relapse-free intervals than those with low or high Ki67%, as determined by flow cytometry. In human medicine, Hall *et al*. [49] analyzed the survival rate of patients with different grades and Ki67 indexes, demonstrating that those affected by LG NHL with high Ki67 indexes had worse survival rates than those with low Ki67 index and that HG NHL with very high Ki67 indexes had better survival rates when compared to those with lower indexes. Therefore, Hoster *et al*. recommended that Ki67 index should be routinely estimated in clinical practice to allow for more accurate individual risk estimation [48].

Some of the described discrepancies between subtypes and proliferation indexes, particularly in the LG T-cell small clear subtype with high Ki67 index, may be related to the process of transformation. As defined by the European canine lymphoma group, “transformation is the evolution of an indolent to an aggressive lymphoma, typically harboring a very poor prognosis” [3]. In veterinary medicine, this phenomenon has been undervalued, although it is a possible indolent lymphomas’ evolution [50]. To the best of authors’ knowledge, however, there were no reports confirming the transformation of canine indolent lymphomas.

Different factors such as genetic modifications, epigenetic transformations, and microenvironmental conditions may contribute to reprograming and transforming the original neoplastic mass into a more aggressive one [50,51]. Furthermore, lifestyle factors such as smoking habits, obesity, and alcohol consumption have been shown to influence the overall survival rate of human NHL patients [52].

The potential links between environmental pollutants and the risk of developing human NHL [24,31] and canine lymphomas [6,20,21,53] have been described. There is, however, a lack of studies relating environmental factors to the biological behavior of canine lymphomas, particularly those with potential prognostic or predictive value.

In our series, albeit small, a statistically significant relationship between exposure to tobacco smoke and the lymphomas’ proliferation, as expressed by the Ki67 index, emerged. High Ki67 indexes were positively correlated with the number of smoker cohabitants. These results are consistent with the hypothesis that second-and third-hand exposure to tobacco smoke could be considered as a hazardous factor for dogs with lymphomas by being associated with enhancement of their proliferative activity or transformation. Yet, it should be noted and highlighted that larger and more controlled studies are warranted to confirm this hypothesis. Given the small sample used, the external validity of this study is not possible.

## Conclusion

In summary, our results suggest that the cytological diagnosis of canine lymphomas benefits from being complemented with immunocytochemical studies that include subtyping and proliferative assessment, both contributing for the prognosis and the therapeutic planning. Furthermore, exposure to tobacco smoke in second-and third-hand ways seems to be related to higher proliferating indexes of canine lymphomas, independently of their subtype or grade.

## Authors’ Contributions

KCP was the research executer and coordinator. MS collaborated in the cytological diagnosis and LLM with sample collection. JNR oriented and supervised data analysis. AJM participated in the study planning, execution, and manuscript revising. All authors read and approved the final manuscript.
